# Photodynamic Treatment of Tumor with Bacteria Expressing KillerRed

**DOI:** 10.1371/journal.pone.0131518

**Published:** 2015-07-27

**Authors:** Libo Yan, Masamitsu Kanada, Jinyan Zhang, Shigetoshi Okazaki, Susumu Terakawa

**Affiliations:** Medical Photonics Research Center, Hamamatsu University School of Medicine, Handayama, Higashi-ku, Hamamatsu, Japan; Justus-Liebig-University Giessen, GERMANY

## Abstract

Photodynamic therapy (PDT) is a cancer treatment modality in which a photosensitizing dye is administered and exposed to light to kill tumor cells via the production of reactive oxygen species (ROS). A fundamental obstacle for PDT is the low specificity for staining solid tumors with dyes. Recently, a tumor targeting system guided by anaerobic bacteria was proposed for tumor imaging and treatment. Here, we explore the feasibility of the genetically encoded photosensitizer KillerRed, which is expressed in *Escherichia coli*, to treat tumors. Using nitroblue tetrazolium (NBT), we detected a lengthy ROS diffusion from the bodies of KillerRed-expressing bacteria in vitro, which demonstrated the feasibility of using bacteria to eradicate cells in their surroundings. In nude mice, *Escherichia coli* (*E*. *coli*) expressing KillerRed (KR-*E*. *coli*) were subcutaneously injected into xenografts comprising CNE2 cells, a human nasopharyngeal carcinoma cell line, and HeLa cells, a human cervical carcinoma cell line. KR-*E*. *coli* seemed to proliferate rapidly in the tumors as observed under an imaging system. When the intensity of fluorescence increased and the fluorescent area became as large as the tumor one day after KR-*E*. *coli* injection, the KR-*E*. *coli*-bearing tumor was irradiated with an orange light (λ = 540 − 580 nm). In all cases, the tumors became necrotic the next day and were completely eliminated in a few days. No necrosis was observed after the irradiation of tumors injected with a vehicle solution or a vehicle carrying the *E*. *coli* without KillerRed. In successfully treated mice, no tumor recurrence was observed for more than two months. *E*. *coli* genetically engineered for KillerRed expression are highly promising for the diagnosis and treatment of tumors when the use of bacteria in patients is cleared for infection safety.

## Introduction

Photodynamic therapy (PDT) is an alternative cancer treatment modality in which a photosensitizing drug is administered to patients and then a light of a specific wavelength is shed to them to kill their tumor cells via the production of reactive oxygen species (ROS) [1]. This treatment has been successfully used in thousands of cancer patients worldwide as a new and promising treatment modality [2, 3]. PDT involves three key components: a photosensitizer, a light source and tissue oxygen [4]. Photosensitizers are reagents that efficiently produce ROS upon light illumination, and are commonly used to study the oxidative stress in basic biological processes or in photodynamic therapy. There are many photosensitizers currently available, such as a porphyrin mixture developed by Schwartz, *i*.*e*., a hematoporphyrin derivative (HpD), Photofrin Ι, Talaporphyin, Laserphyrin, and Visudine. As a precursor of photosensitive protoporphyrin 9, 5-aminolevulinic acid (5-ALA) is also commonly use. These chemical compounds have several limitations including poor reproducibility, limited pharmacokinetics, impurity, and aggregation [5]. All known photosensitizers require that a compound is administered to living individuals exogenously. Systemically administered photosensitizers preferentially accumulate in tumors compared with normal tissue. However, their selectivity may not be sufficiently high; thus, treated patients often suffer from long-term skin sensitivities caused by the retention of photosensitizers in their skin and subsequent exposure to light [6]. This limitation has prompted the search for a new type of photosensitizer.

Recently, KillerRed, a phototoxic red fluorescent protein, has been reported [7]. KillerRed has been demonstrated to be a genetically encoded photosensitizer that kills *Escherichia coli* and eukaryotic cells through the production of reactive oxygen species (ROS) upon light irradiation. The red fluorescent protein KillerRed was engineered from the hydrozoan chromoprotein anm2CP, a homolog of green fluorescent protein (GFP). KillerRed is the first strongly phototoxic protein among the GFP family members. The phototoxicity of KillerRed exceeds that of the other green and red fluorescent proteins by at least 1,000-fold, which is strong enough to kill bacterial and eukaryotic cells through the inactivation of specific proteins by chromophore-assisted light inactivation (CALI) [8]. The absorbance spectrum of KillerRed has a maximum at 585 nm and the fluorescence spectrum an emission peak at 610 nm. The KillerRed protein possesses numerous sulphur-containing amino acids (cysteine, methionine) which serve for a self-protection system against ROS generated upon a light exposure. Therefore, KillerRed efficiently destroys biological tissues by light-induced ROS before being photobleached [9]. As an application of KillerRed to a PDT approach, a recombinant immunophotosensitizer was created by conjugating KillerRed with a humanized monoclonal antibody, and its anti-tumor effectiveness has been demonstrated *in vitro* [10]. KillerRed was also applied *in vivo* to observe the role of ROS in transgenic zebrafish [11] and to show its effects on cell division in *Xenopus* embryos [12]. KillerRed-expressing cells were tested for its cytocidal effect by first implanting them to create a xenograft tumor and subsequently irradiating the tumor in mice [13]. In that research, an efficient way of delivering KillerRed to the tumors is wanted. Therefore, it is necessary and important to develop a new delivery system specific to solid tumors for the practical KillerRed gene therapy.

A number of recent studies revealed that bacteria are capable of targeting both primary tumors and metastases, enabling the noninvasive visualization of tumors by making use of bioluminescence or green fluorescent proteins in mouse tumor models [14, 15]. It has been demonstrated that bacteria are naturally capable of homing to tumor sites when systemically or locally administered, resulting in a high level of local replication [16, 17]. These studies prompted us to hypothesize that *E*. *coli* may be used as a gene delivery system for KillerRed targeting to tumors. Furthermore, a photosensitizer genetically encoded in bacteria would be amplified *in vivo* if the bacteria grow in the tumor site, promising a great enhancement of the PDT effect. On the other hand, a question remains whether a ROS produced inside the bacterial body is effective enough to kill tumor cells nearby. Here, we demonstrate a strong eradicating effect of KillerRed-expressing bacteria on the subcutaneously formed tumors of CNE2 and HeLa cells in mice.

## Materials and Method

### Animal model

Five- to six-week-old female BALB/c athymic nude mice (18–23 g body weight) were purchased from the Shizuoka Lab Animal Center (SLC) in Japan. This study was carried out in a strict accordance with the recommendations in the Guide for the Care and Use of Laboratory Animals of the National Institutes of Health. The protocol was specifically approved by the Committee on the Ethics of Animal Experiments of Hamamatsu University School of Medicine (Permit Number: H23-036). All surgery was performed under sodium pentobarbital anesthesia, and all efforts were made to minimize suffering. Animal anesthesia was performed using pentobarbital sodium (10%) at a concentration of 25 mg/kg for PDT and 30 mg/kg for surgery.

### Cell lines and tumors

Poorly differentiated human nasopharyngeal carcinoma CNE2 cells (purchased from cell bank of XiangYa Central Experimental Laboratory of Central South University, China) and a strongly adherent human cervical carcinoma HeLa cells (line 15S3D) were used. CNE2 cells were cultured in high-glucose Dulbecco’s modified Eagle medium (DMEM) containing 10% fetal bovine serum (FBS) and 1% penicillin-streptomycin at 37°C in a 5% CO_2_ atmosphere. HeLa cells were cultured in RPMI 1640 supplemented with 10% FBS (Invitrogen), 2mM glutamine (Invitrogen), 10 U/ml penicillin and 10 μg/ml streptomycin. CNE2 cells (5 × 10^6^) suspended in 0.2 ml of PBS were subcutaneously (s.c.) implanted into the right abdomen of one group of mice. HeLa cells (5 × 10^6^) suspended in 0.2 ml of PBS were s.c. implanted into the right abdomen of another group of mice. After seven days, the volume of HeLa tumor reached about 100 mm^3^. The tumor volume was measured with a caliper, and calculated using the following formula: V = (L × H × W) / 2, where L is the length, W the width, and H the height of the tumor in millimeter [15].

### Transformation of the KillerRed plasmid DNA, KillerRed and intratumoral injection of KR-*E*. *coli*

Briefly, DH5α *E*. *coli* cells (TaKaRa, Tokyo) were transformed with a KillerRed expression vector (cat. #FP963, Evrogen, Moscow) under the control of T5 promoter/lac operator by exposing them to a temperature shock of 42°C after incubation on ice. After incubation in SOC-medium [18] at 37°C for 1 h, they were plated on agar overnight. On the next day, KillerRed was expressed in β-galactosidase of *E*. *coli*. The *E*. *coli* colonies efficiently expressing KillerRed were selected, and amplified in 2 ml of LB medium for overnight on a shaker at 37°C to obtain strongly phototoxic *E*. *coli* as described by Mueller [19]. The mobility of the *KR-E*. *coli* was then confirmed under a fluorescence microscope. *E*. *coli* expressing KillerRed (1 × 10^8^–1 × 10^9^) were collected by centrifugation, and directly injected into the subcutaneous tumor tissue at its base using an insulin syringe (1-cc; Terumo, Tokyo).

### Photodynamic treatment (PDT)

PDT irradiation to the tumors with orange light (λ = 540 − 580 nm) was performed on the next day after *E*. *coli* injection into the tumor. The phototoxic effect were examined in small CNE2 tumors (volume ≈ 80 mm^3^), large CNE2 tumors (volume = 150 mm^3^), and HeLa tumors (volume = 90 − 100 mm^3^). Mice with CNE2 tumors of similar size were randomly divided into three groups (n = 12 in each group): Group A received an intratumoral administration of bacteria expressing KillerRed at a dose of 10^8^–10^9^ bacteria per tumor, and was kept in the dark room overnight. On the next day, tumors, completely filled with bacteria expressing KillerRed, were irradiated with light from an optical fiber connected to a mercury lamp equipped with a band pass filter (540 − 580 nm in wavelength) and an IR-suppression filter for 30 min (fluence = 324 W/cm^2^: measured with a laser power meter, see below). This regimen was chosen following initial optimization experiments. The power of the mercury lamp was measured with a laser power meter (AA30, Scientech Astral, Colorado, USA) before and after the illumination. The tip of optical fiber used for irradiation was mounted above the tumor perpendicular to the animal. The area of irradiation was set so as to cover the entire tumor. During the irradiation, the tumor temperature was measured at its surface by an IR thermograph (CEM-Thermo Diagnostics, CEM-Technology, Nizhniy Novgorod, Russia). Group B (control group I) was subjected to the same irradiation protocol but with the bacteria which did not express KillerRed; Group C (control group II) was subjected to the same irradiation protocol without employing any bacteria in the injection medium. After irradiation, the mice were kept in a dark box for overnight, and then subjected to intermittent measurements for their tumor sizes.

### Fluorescence imaging and quantitative evaluation of fluorescence intensity *in vivo*

To image the fluorescence of KR-B, anesthetized animals were placed in the light-tight chamber of an in vivo imaging system (Xenogen IVIS100 Spectrum, Caliper, Hopkinton, MA, USA), equipped with a cooled charge coupled detector (CCD) camera. The fluorescence emitted from KillerRed-expressing bacteria was collected. All fluorescence images were acquired with 5-second exposure time. Serial fluorescence images were acquired. The fluorescence intensities in these images were quantified and analyzed by using ImageJ 1.43u software (National Institutes of Health, USA). A region of interest (ROI) was selected manually over the fluorescent area. The area of the ROI was kept constant, and the intensity of fluorescence was quantitated from digitized values in the ROI.

### Histological observations

For the histological analysis, two mice were selected separately and randomly from group A, B and C and were sacrificed 24 hours after the irradiation. A portion of each tumor was excised and fixed with 4% formaldehyde (Wako, Osaka) at pH 7.0. After paraffin embedding, a series of 2 μm sections were prepared for each specimen, and mounted on glass slides. The slides were observed for their phototoxic damages under a light microscope after staining with hematoxylin and eosin (H&E) solutions.

### Detection of superoxide released from KR-*E*. *coli* by the NBT method

To monitor O_2_^−^ in the environment of bacteria that expressed KillerRed intracellularly, nitroblue tetrazolium (NBT; Sigma, St Luois, MO) [20] was employed. A 300 μl of KR-*E*. *coli* suspension was placed in a pool (volume = 400 μl) made in a 1.5% agarose (Sigma) gel containing NBT at 0.75 mM in a 35 mm dish. The KR-*E*. *coli* solution was placed in the pool in the center of the dish, and irradiated with a light from a mercury lamp equipped with a band pass filter (540 − 580 nm) for 30 min [7]. When NBT is reduced by superoxide, it turns from a colorless compound into a blue formazan precipitate. Thus, the O_2_^-^ activity from KillerRed was evaluated by monitoring the precipitation of the blue-colored formazan in the gel phase. The bacterial invasion into the agar gel was assumed to take place only at a very slow rate.

### Measurement of singlet oxygen

For examination of the singlet oxygen production, a near-infrared light emitted from singlet oxygen was directly measured using a photomultiplier-based apparatus (NIR-PII System, Hamamatsu Photonics K.K., Hamamatsu, Japan) [21,22]. The emission from singlet oxygen (λ = 1,270 nm) was detected using an infra-red-gated image intensifier after a passage through a polychrometer (250is, Chromex, NM, USA). Signals were accumulated by a repeated gate operation (>300 times), and then averaged. The calibration of wavelength was performed using a spectral calibration lamp (Krypton type, Oriel Instruments, CT, USA).

Singlet oxygen decay curves were monitored using a photo detector (Spectra-Physics, CA, USA). The excitation wavelength was 585 nm. Emission was detected using a photomultiplier (R5509-42, Hamamatsu Photonics K.K., Hamamatsu, Japan) combined with a monochrometer (HR-320, Jobin Yvon, France). Data were stored in a multichannel scaler (SR430, Stanford Research Systems, CA, USA). All measurements were performed at 22°C. The absorption spectra were measured before and after decay measurements to monitor the photobleaching of the dye.

### Statistical analysis

Data are shown as the mean ± SEM for experiments performed *in vitro* studies or *in vivo* studies. In statistical significance testing, *p* values were calculated using the *t*-test.

## Results

### Superoxide released from KR-*E*. *coli* upon irradiation

**Fig 1:**
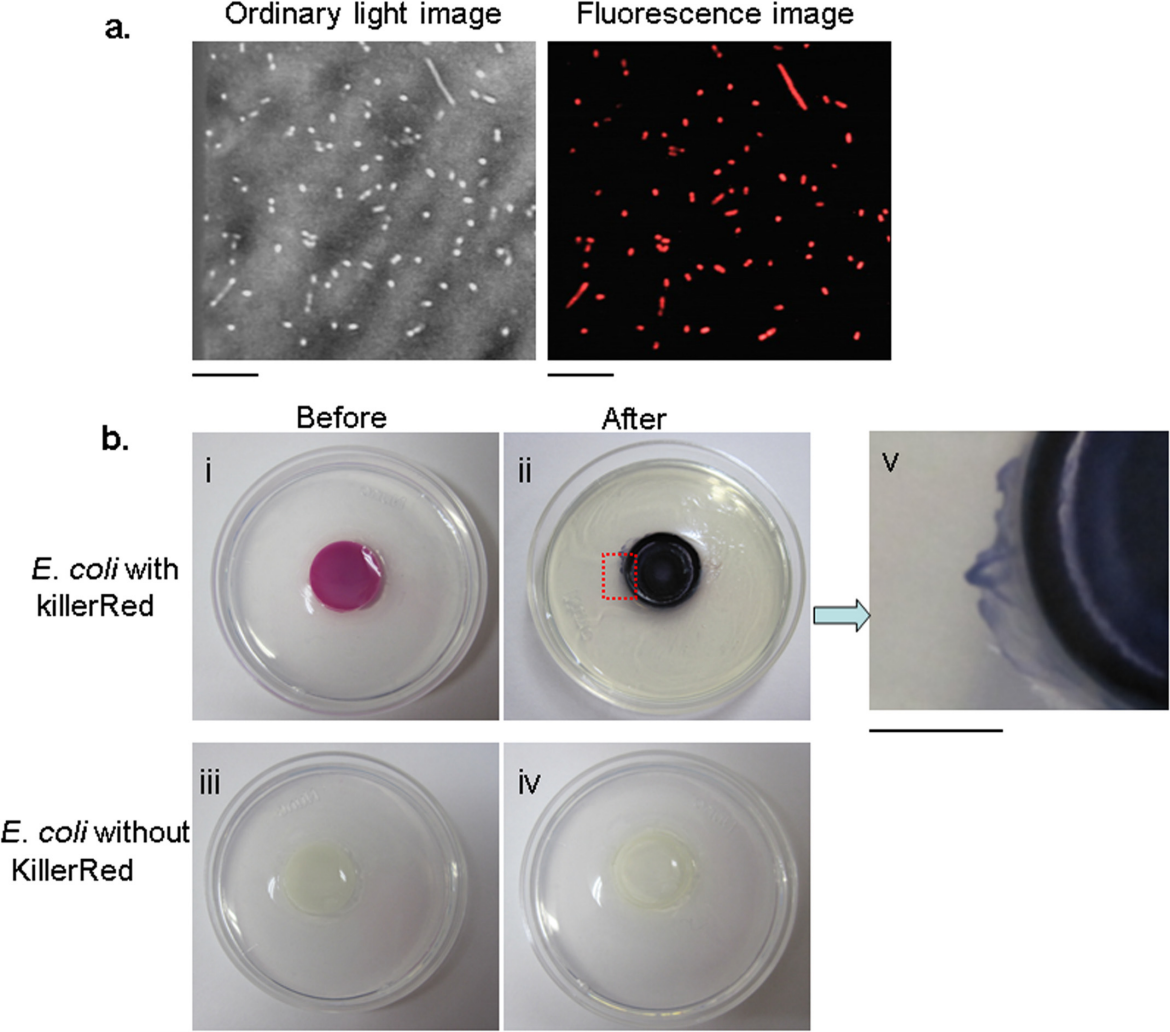
Superoxide is released from KR-*E*. *coli* upon orange light irradiation. **a,** Ordinary light image (left) and fluorescence image (right) of a KR-*E*. *coli* suspension observed under a microscope. Bar, 10 m. **b,** The production of superoxide by the irradiation of KR-*E*. *coli* detected with nitroblue tetrazolium (NBT) in an agarose gel. The top panels demonstrate a change in the *E*. *coli* with KillerRed before and after irradiation. A magnified portion (right) of the agarose gel near the KR-*E*. *coli* containing central pool indicates a formazan precipitation due to the diffusion of superoxide from the pool. Bar, 5 mm. The bottom panels demonstrate change in the color of the *E*. *coli* without KillerRed expression before and after irradiation.

In a KR-*E*.*coli* suspension, strong red fluorescences were observed only in the bacterial cell bodies. The fluorescence from KillerRed that might leak from the bacterial body to surrounding solution was below the detection level (Fig 1A). This observation led us to the question whether ROS created by KillerRed inside the bacterial body could reach the tumor cells to provoke the phototoxicity. To visualize the diffusion of superoxide produced by KillerRed upon irradiation, an NBT-containing gel was used. Prior to irradiation, the color of the KR-*E*. *coli* suspension in the central pool of the dish was pink when viewed under ordinary laboratory light. However, after the orange light irradiation, the color of the bacteria suspension drastically changed to deep purple, indicating an efficient production of a formazan precipitate (Fig 1B (i, ii)). In the gel phase marginal to the central pool, formazan precipitate was also observed (within 30 min) at a distance greater than 2 mm from the edge to the central pool. A magnified image of the gel plate surrounding the bacteria-containing pool demonstrated that a sufficient amount of superoxide diffused into the gel phase. In this way, the diffusion of O_2_^−^ from the central pool to the surrounding agar gel was demonstrated. In contrast, in the suspension in the pool containing bacteria without KillerRed (control), no color change was observed in the central pool as well as in the surrounding gel upon irradiation (Fig 1B (iii, iv)).

### Singlet oxygen is not produced from KillerRed upon irradiation

**Fig 2:**
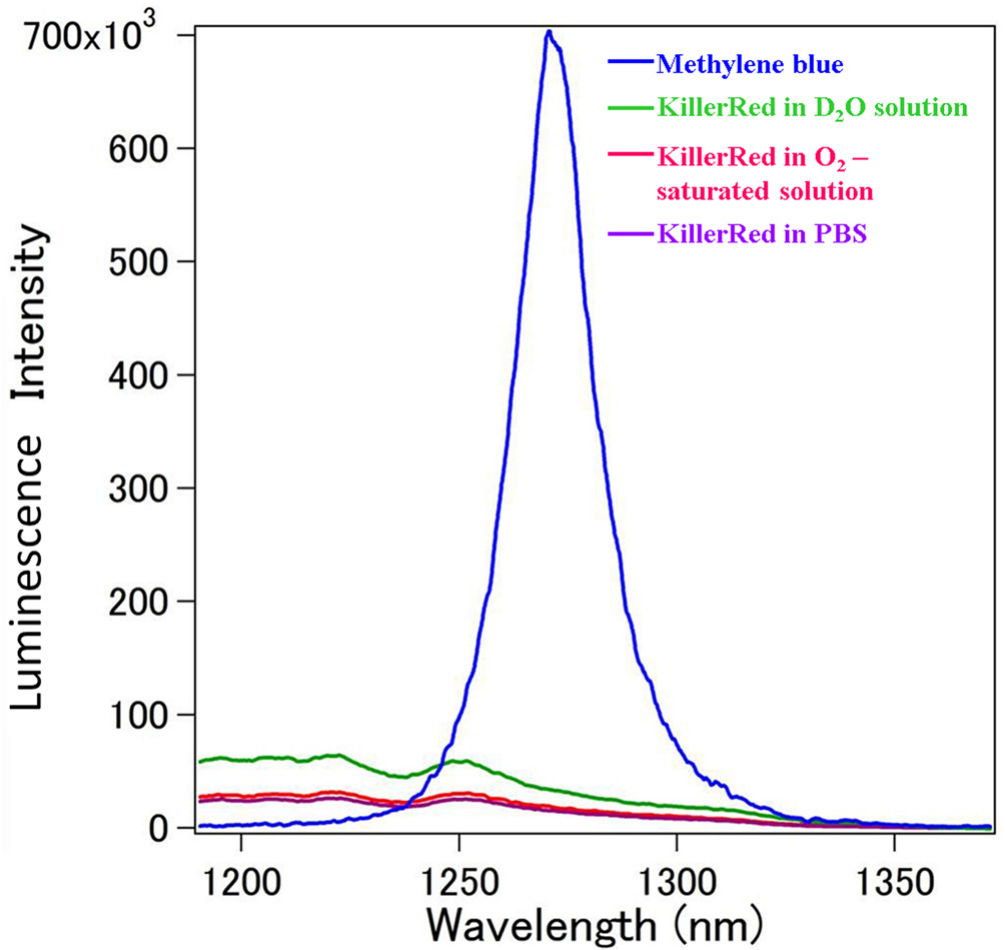
Absence of singlet oxygen in the phototoxic effect of illuminated KR-*E*. *coli*. To quantitatively measure the singlet oxygen produced by KillerRed, the intensity of spontaneous luminescence was measured at wavelengths in a near infrared region. (1) KillerRed in PBS. (2) KillerRed in an O_2_-saturated solution. (3) KillerRed in a deuterium oxide (D_2_O) solution. (4) Methylene blue solution used as a control. In reference to methylene blue, KillerRed did not produce any singlet oxygen upon irradiation.

For quantitative estimations of the singlet oxygen produced by KillerRed, the spontaneous emission (luminescence) at the wavelength of 1,270 nm was measured when KillerRed was in a PBS solution, an O_2_^−^ saturated solution, and a deuterium oxide (D_2_O) solution. A methylene blue solution was used as a control. In reference to the methylene blue solution, the KR*-*containing solution did not produce any singlet oxygen upon irradiation (Fig 2), solidly confirming previous observations [23, 24]. A forced saturation of test solution with O_2_ gas did not enhance the peak of spectrum at 1,270 nm at all. D_2_O is known to extend a lifetime of−O_2_ and thus increases the efficiency of singlet oxygen-generation from photosensitizers [25, 26]. However, the replacement of 50% of water in the KillerRed-containing solution with deuterium oxide did not promote the singlet oxygen production at all.

### Intratumoral proliferation of KR-*E*. *coli*

**Fig 3:**
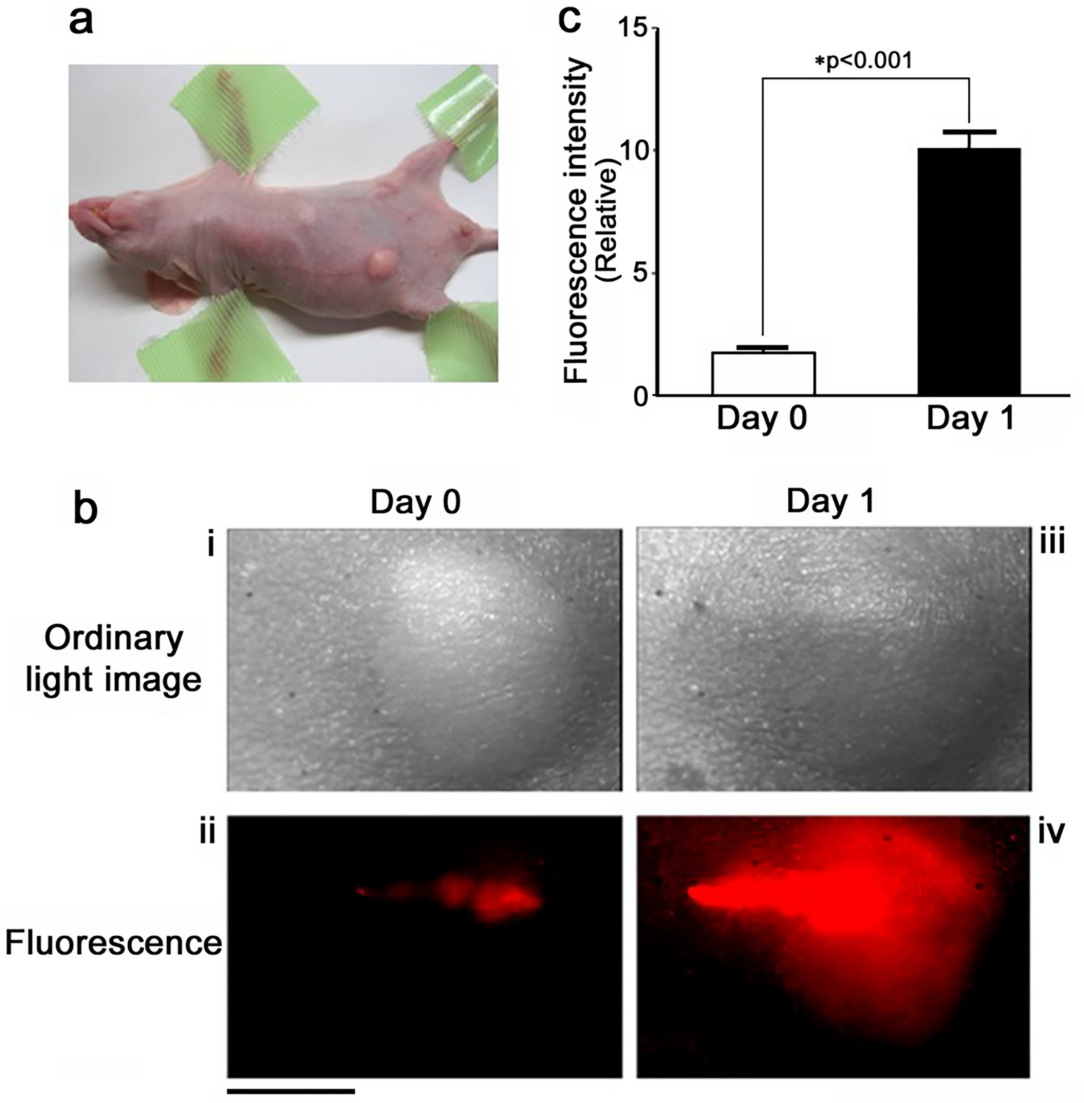
Intratumoral proliferation of KR-*E*. *coli*. **a,** NPC tumor subcutaneously formed in a nude mouse. **b,** The distribution of KR-*E*. *coli* immediately after intratumoral injection (left), and one day after the injection (right). Scale bar, 5 mm. **c,** Quantification of the fluorescence intensity of KR-*E*. *coli* immediately after the intratumoral injection and one day after the injection (n = 10). *p<0.001 (*t*-test). Error bars indicate standard error of the mean (SEM). The fluorescence intensity was calculated by summating the digital values of all points in the field of view, and expressed in a relative manner.

In the transient expression system, KR-*E*. *coli* did not retain the plasmid DNA in the absence of appropriate antibiotics, thus, we firstly examined how long the intratumorally injected KR-*E*. *coli* could survive and maintained KillerRed expression *in vivo*. The KR-*E*. *coli* collected by centrifugation were directly injected into subcutaneous tumor formed by CNE2 cells in athymic nude mice (Fig 3A). Immediately after the intratumoral injection of KR-*E*. *coli*, the intensity and the area of red fluorescence of bacteria were weak and small (Fig 3B (ii)). The mice were subsequently maintained in a dark room for overnight. On the next day, the intensity and the area of fluorescence in the tumor became larger enough to occupy the entire tumor (volume ~50 mm^3^) even in the absence of antibiotics *in vivo* (Fig 3B (iv)). A quantitative evaluation demonstrated that the fluorescence intensity of KillerRed in the tumor became approximately five-fold higher after 24 h (Fig 3C). Subsequent monitoring also revealed that the fluorescence intensity in the tumor peaked at Day 1, and decreased thereafter to an undetectable level in one week. It is proven that the intensity of fluorescence correlated directly with the number of bacteria [14]. Therefore, these results suggested that the intratumorally injected KR-*E*. *coli* survived in the tumor and kept KillerRed expression as well for at least one day without antibiotics administration. It was likely that the bacteria proliferated in the tumor for a short period of time. Based on those observations, the optimal time for the PDT of tumors was determined to be the next day after intratumoral injection for the following experiments.

### Phototoxicity of intratumoral KR-*E*. *coli* on the subcutaneously formed CNE2 and HeLa tumors

**Fig 4:**
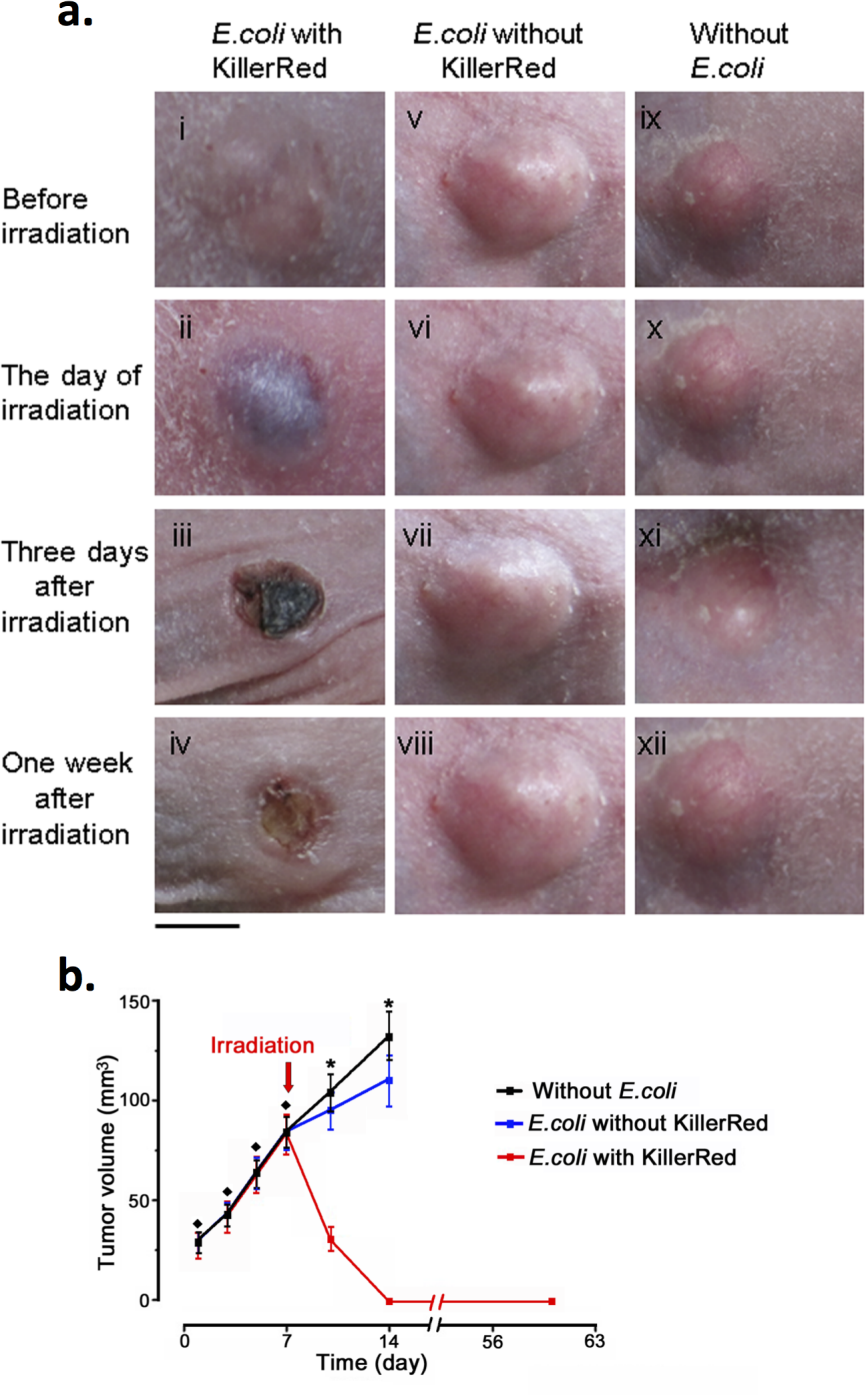
Effect of PDT with KR-*E*. *coli* on CNE2 tumors. **a,** The left column represents the tumor (CNE2) injected with KR-*E*. *coli* and irradiated with the orange light. The middle column represents the tumor injected with wild type *E*. *coli* and irradiated with the orange light. The right column represents the tumor irradiated without *E*. *coli* injection (n = 12). Bar, 5 mm. **b,** Growth curves of tumors in the presence and absence of KillerRed-*E*. *coli*. The black line indicates the volume change of tumors irradiated without *E*. *coli*. The blue line indicates the volume change of tumors injected with *E*. *coli* that expressed no KillerRed. The red line indicates the volume change of tumors injected with *E*. *coli* that expressed KillerRed. All tumors were irradiated at the same dose on the day 7 after tumor cell transplantation (Day 1 after bacterial injection). Before treatment: ^♦^p > 0.05 (rank sum test). After treatment: *p < 0.01 (rank sum test). Error bars indicate SEM.

After the treatment with the orange light, only mice with tumors bearing KR-*E*. *coli* were affected (Fig 4A (i-iv)). The color of the tumors became purple as maculae and scabs were formed in the following days. All of the tumors into which KR-*E*. *coli* was injected gradually disappeared after the irradiation, and the skin was healed after one week (Fig 4A (iv)). In contrast, normal tissues near the tumors demonstrated no noticeable change before and after the irradiation. Moreover, two other control groups (tumors with *E*. *coli* expressing without KillerRed and tumors lacking *E*. *coli*) demonstrated no change after the treatment compared with the KR-*E*. *coli*-injected tumors (Fig 4A (v-xii)). These results suggest that KR-*E*. *coli* has a strong phototoxic effect on the tumors. Tumors injected with KR-*E*. *coli* and treated with irradiation demonstrated no sign of recurrence in the following two months (Fig 4B).

**Fig 5:**
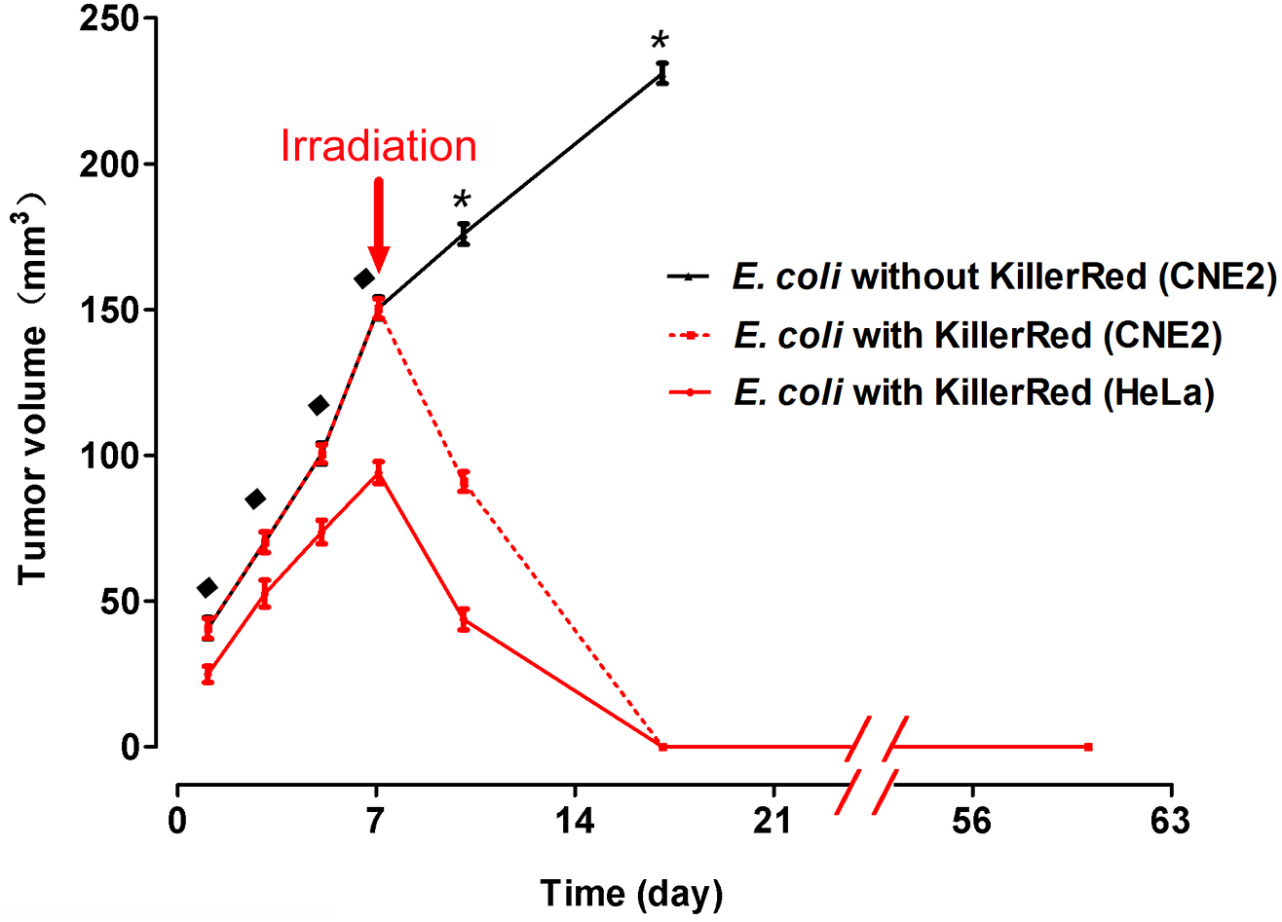
Growth curves of the HeLa tumors and CNE2 tumors of a large size treated with KR-*E*. *coli*. The black line indicates the volume change of tumors injected with *E*. *coli* that expressed no KillerRed. The red dotted line indicates the volume change of CNE2 tumors (volume = 150 mm^3^) injected with *E*. *coli* that expressed KillerRed. The red full line indicates the volume change of HeLa tumors injected with *E*. *coli* that expressed KillerRed. All tumors were irradiated at the same dose on the day 7. Before treatment, ^♦^p > 0.05 (rank sum test), error bars indicate SEM. After treatment, *P< 0.01 (rank sum test), error bars indicate SEM.

We further examined the phototoxicity of KR-*E*. *coli* on the well-developed large CNE2 tumors (approximate volume of tumors reached 150 mm^3^) and HeLa tumors, which were regarded as difficult to completely cure using a single PDT regimen [27]. After an irradiation, we observed a similar change in the well-developed tumors. The scabs formed on the surface of the tumors were large enough to cover entire tumors. One week after the treatment, all the CNE2 tumors (N = 6 out of 6) and some HeLa tumors (N = 4 out of 6) were completely eradicated (Fig 5). In two mice with HeLa tumors, the internal bleeding did not occur even after irradiation. It is likely that the irradiation light failed to reach the KR-*E*. *coli* located inside the tumors. When there was neither photobleaching nor internal bleeding observed in the tumor, the PDT failed. Otherwise, all cases examined were successful and the tumors were thoroughly eradicated.

### Histological analysis of phototoxicity induced by KR-*E*. *coli* on CNE2 tumors

**Fig 6:**
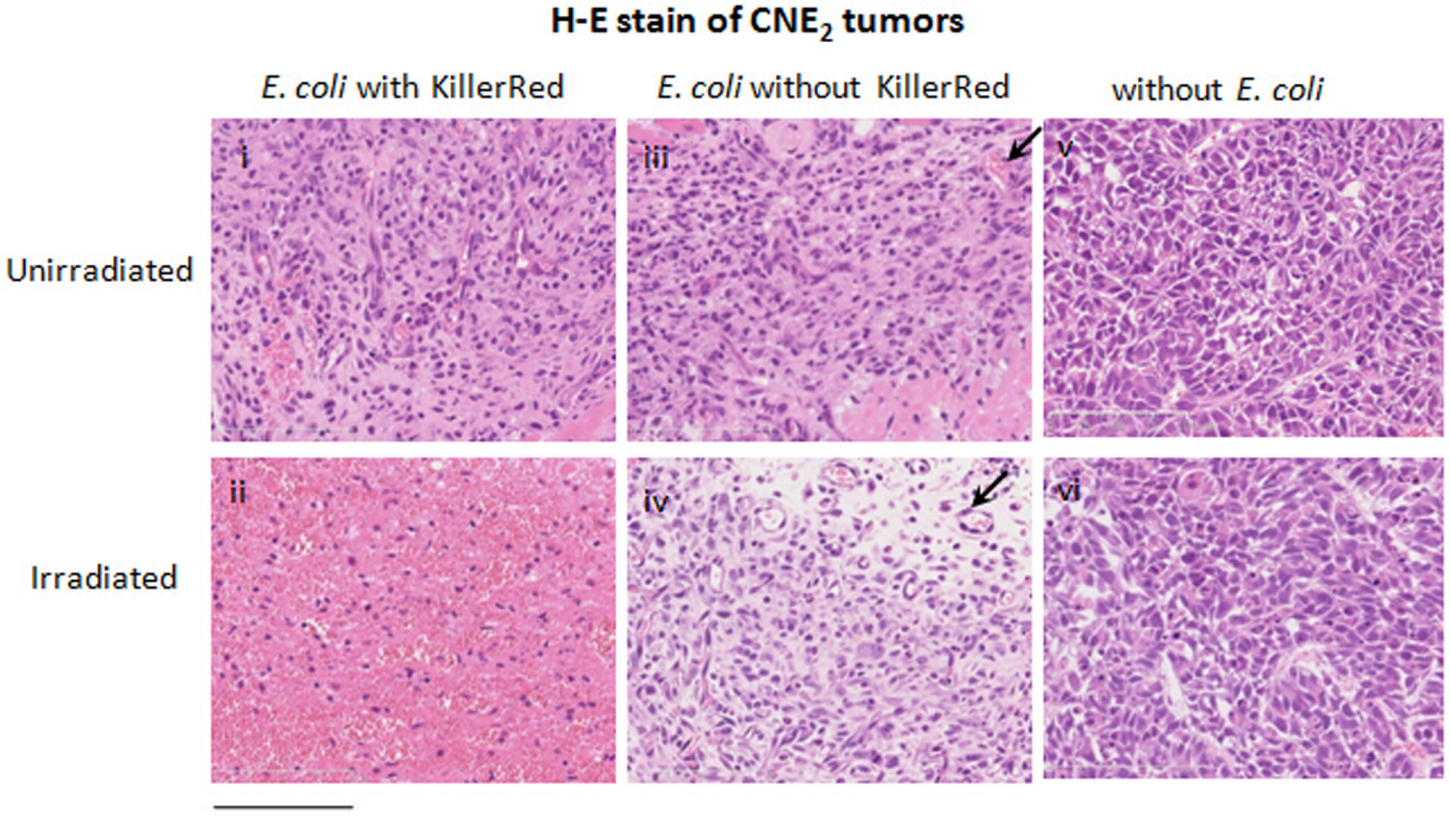
Histological sections of tissue showing the phototoxicity induced by KR-*E*. *coli* on CNE2 tumors. The top panels demonstrate sections of CNE2 tumors that were injected with KR-*E*. *coli*, *E*. *coli* without KillerRed expression, and a physiological saline, and fixed before irradiation. The bottom panels demonstrate the preparations treated in the same way but fixed after irradiation with the orange light. All sections were H&E stained. The arrows indicate blood vessels of the tumor. Bar, 200 m.

We next performed a histological analysis of tumor tissues obtained before and after irradiation with KR-*E*. *coli* (Fig 6). There were a few *E*. *coli* expressing KillerRed and few necrotic tumor cells scattered in the tumor tissue prior to PDT (Fig 6 (i)). However, there were many dead cells near the *E*. *coli* expressing KillerRed, and extensive cellular degeneration was observed in tumors 24 h after PDT (Fig 6 (ii)). The cells were stained strongly with eosin, half amorphous, and not clearly outlined. The number of nuclei stained in a field of view was much smaller than that in control. After the treatment, blood vessels were severely destroyed by ROS. Many red dots in the histological section indicated the erythrocytes leaked from the vessels damaged by PDT. A similar view was rarely observed in tumor tissue injected with *E*. *coli* of no KillerRed expression (Fig 6 (iv)). In another control group observed without any injection of E. coli, there was no noticeable change before and after PDT (Fig 6 (v, vi)).

## Discussion

In this study we have firstly demonstrated that KR-*E*. *coli* can be successfully used to kill primary human-originated cancer cells as a target of bacteria delivered gene expression system in photodynamic treatment *in vivo*. The phototoxicity of intratumoral KR-*E*. *coli* was demonstrated to be high enough to eradicate all CNE2 tumors and some HeLa tumors to the extent that no recurrence took place for more than two months leaving the tested mice under a good growing condition. Histological data have also demonstrated that irradiation of bacteria expressing KillerRed in mice leads to a complete damage of the tumor tissues. These results suggest that KillerRed can be useful as a genetically encoded potent photosensitizer for the photodynamic therapy (PDT) of cancer, and that it may serve as a model protein of bacteria-mediated gene delivery system.

KillerRed, as a genetically encoded photosensitizer, is different from chemically synthesized photosensitive dyes in some respects. Therefore, it has some advantages over other photosensitizers prepared by chemical means. Firstly, the fluorescence intensity of KillerRed can be greatly amplified as the host bacteria may proliferate *in situ*. This amplification of photosensitizing power is unique to the genetically encoded photosensitizer. Secondly, superoxide is produced directly from the KillerRed. We concerned about the efficient release of ROS from the KillerRed as a necessary property to induce cytotoxic effects in tumor cells. Our results obtained by using NBT and direct near-infrared light measurements have demonstrated that superoxide is released from KillerRed but singlet oxygens is not, indicating that KillerRed acts as a type I photosensitizer. Type I or type II process depends on the nature of the primary steps, namely, the initial involvement energy transfer from a photoexcited photosensitizer to superoxide or hydroxyl radical. The superoxide which has a life time much longer than that of singlet oxygen travels for a long distance by the diffusion. This is probably the reason why the ROS generated only in the bacterial bodies can kill tumor cells in the neighborhood. Our results are consistent with the observations described in previous reports [10, 23]. Thirdly, KillerRed has a lower phototoxicity than other organic photosensitizers when compared in a molecular unit [7]. Even when KillerRed-expressing bacteria non-specifically accumulate in the skin, mucosa and so on, the side effects of their light exposure would be minimal.

A challenge in the current PDT approaches is a specific delivery of photosensitizers [28–30]. Certain species of anaerobic bacteria are localized and specifically germinate in the hypoxic and necrotic regions of tumors as tumor-specific vectors for the delivery of antitumor genes by systemic application [31, 32]. However, they would not grow in well-oxygenated normal tissues [33–35]. Therefore bacteria expressing KillerRed become an ideal delivery vector for an anticancer gene product to solid tumors, which was wanted by a research group of Shirmanova [13].

The present study demonstrated that KR-*E*. *coli* may proliferate in tumor tissues and effectively kills human tumor cells upon irradiation after intratumoral injection. This amplification by themselves is another important characteristic of bacteria [36]. In the initial part of our study, it was demonstrated that because of the overnight proliferation of transiently transformed KR-*E*. *coli* after intratumoral injection *in vivo*, the fluorescence intensity of KR-*E*. *coli* increased nearly five times relative to baseline (Fig 3). At the same time, the bacteria could hold the plasmid DNA and maintained its protein expression *in vivo* even without antibiotics treatment for at least two days. This feature allowed us to examine the phototoxicity of subcutaneously injected KR-*E*. *coli* without establishing the bacteria that stably express KillerRed.

There are numerous routes for administration of the photosensitizer such as the intravenous injection and oral medications and so on [2]. However we used direct intratumoral injection in our study. This method has advantages, such as direct and effective killing tumor cells upon irradiation and avoiding side-effects including undesirable surrounding tissue necrosis [37]. Our results have demonstrated that KR-*E*. *coli* effectively kills all tumors, and that no recurrence was observed for two months (Fig 4B). Moreover, all mice of the test group were healthy after the treatment. However, this treatment also had some limitations. For example, the bacteria can not target metastatic tumors located remotely from the injected site or small satellite tumors under therapy. If we could administer KR-*E*. *coli* from the tail vein, probably the bacteria could target both the original and metastatic tumors, and further kill all tumors *in vivo* when irradiated. As a matter of fact, sending the bacteria to the tumor site from the tail vein was not easy, because the bacteria in the vein were cleaned off by the remaining immune system. This route of administration should be developed for future application.

The KR-*E*. *coli* PDT mechanism is still unclear. From the H&E staining histology (Fig 6), many tumor cells were obviously considered to be damaged by irradiation. Such a change was more obvious in the test group with KR-*E*. *coli* than in control group of *E*. *coli* without KillerRed. The main difference between above two groups was KillerRed expression. Therefore, we suppose that the superoxide production from KR-*E*. *coli* is the major mechanism of this treatment. Besides the direct damage of tumor cells caused by superoxide, the vascular damage is also a possible mechanism of KR-*E*. *coli* PDT. The color of tumor surface obviously changed to bluish because of the subcutaneous bleeding after irradiation (Fig 4A (ii)). At the same time, many red dots appeared in a scattered form in the histological sections, indicating an erythrocyte leakage out of vessels. The nature of such changes was strong vascular reaction resulting in a hemorrhage. It is solidly concluded that, after the treatment, blood vessels were severely destroyed by superoxide produced by KR-*E*. *coli*, and that this circulatory disorder might be one of the reasons for the tumor cell death. As for the control with *E*. *coli* that produced no KillerRed, there was a small difference in damage between the group before treatment and that after it (Fig 6 (iii) and 6 (iv)). It might be also possible that bacterial lipopolysaccharide (LPS) killed the tumor cells by serving as an adjuvant in a manner similar to inflammatory cytokines released from macrophages [38]. Thus, bacteria alone did have a small growth-inhibiting effect on tumors.

## Conclusion

In the near future, a method would probably be developed for dual use of KillerRed for identification and treatment of metastatic foci by systemically administered bacteria that express the genetically encoded photosensitizer. At the moment, the present approach of PDT awaits confirmation of low side effects due to the bacterial infection. A high sensitivity of bacteria to proper antibiotics should be confirmed also to prevent undesirable infectious complications. Once the safety issues are sufficiently addressed, our approach would be useful for human metastatic tumors, which will open new perspectives for photodynamic therapy.
